# Repeated superovulation may affect mitochondrial functions of cumulus cells in mice

**DOI:** 10.1038/srep31368

**Published:** 2016-10-04

**Authors:** Juan-Ke Xie, Qian Wang, Ting-Ting Zhang, Shen Yin, Cui-Lian Zhang, Zhao-Jia Ge

**Affiliations:** 1 Institute of Reproductive Medicine, Henan provincial People’s Hospital & People’s Hospital of Zhengzhou University, Zhengzhou, Henan, P.R. China; 2 Institute of Reproductive Sciences, College of Animal Science and Technology, Qingdao Agricultural University, Qingdao, Shandong, P.R. China

## Abstract

Controlled ovarian stimulation by exogenous gonadotrophins is a key procedure during the *in vitro* fertilization cycle to obtain a sufficient number of oocytes in humans. Previous studies demonstrated that repeated superovulation had deleterious effects on the ovaries. However, whether repeated superovulation adversely affects the mitochondrial functions of cumulus cells remains unclear. In this study, mice were divided into three groups: superovulation once (R1); superovulation three times (R3), and superovulation five times (R5). We evaluated the effects of repeated superovulation on mitochondrial DNA copies (mtDNA) and observed decreased mtDNA copies per cell with increasing number of superovulation cycles. Further, we investigated the DNA methylation status in exon 2 and the mRNA expression level of nuclear-encoded DNA polymerase gamma A (*PolgA*). The results showed that the DNA methylation levels of *PolgA* in R1 and R5 were slightly lower than in R3. Additionally, the altered DNA methylation in *PolgA* coincided with the changes in *PolgA* expression in cumulus cells. We also found that the mRNA expression of *COX1*, *CYTB*, *ND2*, and *ND4* was altered by repeated superovulation in cumulus cells. Thus, repeated superovulation had adverse effects on mitochondrial function.

Assisted reproductive technology (ART) is a highly successfully and widely used method for the treatment of subfertility/infertility. In 2013, ART contributed to 1.6% of all infants in the United States^1^. ART cycles were performed in a total of 285 million inhabitants in Europe in 2011^2^. Over the past decade, the number of babies born by ART has exponentially increased and now accounts for 2–5% of infants in developed countries^3^. Thus, the future health of ART infants has been widely discussed because germ cells and early embryos are sensitive to the environment^4^,^5^. Scherrer *et al*.^6^ reviewed the association between ART and cardiovascular dysfunction in children. Previous studies reported that artificial manipulations during ART altered the epigenetic modifications of embryos and placentas^7^,^8^,^9^.

High-dose exogenous gonadotrophins are used in ART to retrieve a sufficient number of oocytes from humans and animals. In the clinic, a number of women undergo several ART procedures before successful delivery. Thus, these women experience repeated ovary stimulation by exogenous gonadotrophins. A previous study demonstrated that DNA methylation of imprinted genes, besides H19, was not affected in mouse oocytes, although a large amount of exogenous gonadotrophin was used^10^. Market-Velker *et al*. suggested that the methylation of imprinted genes in embryos was altered by gonadotrophins in a dose-dependent manner^7^. However, another study indicated that embryonic imprinting perturbation is not induced by superovulation^11^. These studies suggest that superovulation has no significant effects on the methylation patterns of imprinted genes in oocytes, but oocyte quality may be compromised.

Repeated ovarian stimulation induced oxidative damage and mitochondrial DNA (mtDNA) mutation in mouse ovaries^12^. Additionally, repeated superovulation affects ovarian structure and function in rhesus monkeys^13^. Multiple superovulation phases changed the organelle distribution in mouse oocytes^14^. These data indicate that repeated superovulation reduces oocyte quality, but the mechanism remains unclear. Oocyte development competence is associated with the surrounding cumulus cells^15^. Cumulus cells supply energy substrates and essential nutrients to the oocyte during oogenesis^16^. Mitochondria are the primary organelles that supply energy and metabolic substrates in most eukaryotic cells. Thus, we hypothesized that repeated superovulation may adversely affect mitochondrial functions in cumulus cells. In this study, we investigated the effects of repeated superovulation on mtDNA copy number in cumulus cells. mtDNA copy number is regulated by mitochondrial-specific DNA polymerase γ (POLG)^17^,^18^. The activity of POLG is controlled by the catalytic subunit A (POLGA)^19^, whose expression is regulated by DNA methylation patterns in exon 2^20^. Thus, we investigated the DNA methylation status in exon 2 of *PolgA*. Additionally, we determined the expression of cytochrome b (CYTB), cytochrome c oxidase subunit 1 (COX1), NADH dehydrogenase subunit (ND4), and NADH dehydrogenase subunit 2 (ND2), which are encoded by mtDNA.

## Results

### Repeated superovulation reduced mtDNA copies per cumulus cell

**Figure 1:**
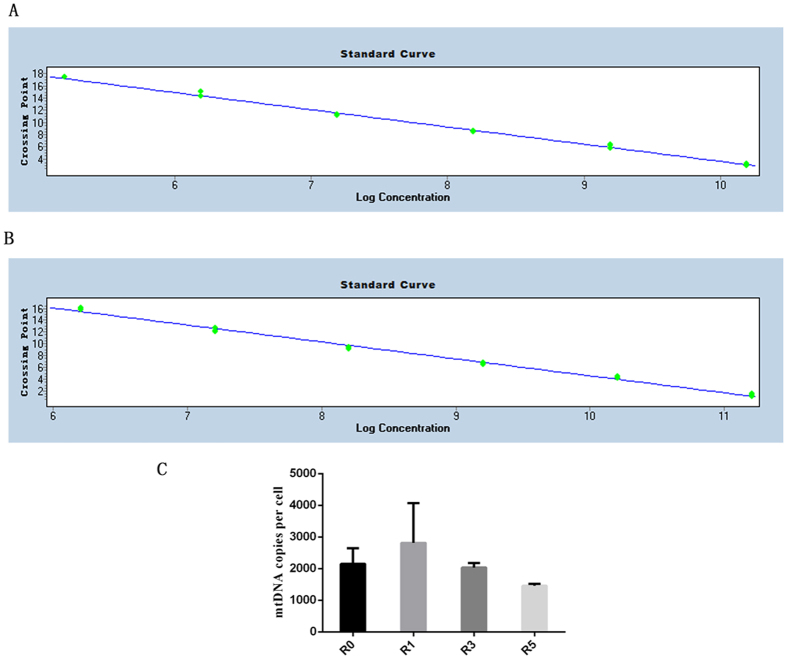
Mean mtDNA copy numbers in cumulus cells. The standard curve of β-actin and mtDNA fragment was produced by real-time PCR in (**A**,**B)** the mean mtDNA copies per cell was evaluated and is shown in (**C**). R1 (n = 12), one superovulation; R3 (n = 12), three superovulations; R5 (n = 12), five superovulations; **represents P < 0.01.

β-actin and the mtDNA fragment were generated by real-time PCR, and a standard curve was drawn (Fig. 1A,B). Next, we determined the number of mtDNA copies per cell and found that repeated superovulation reduced the mean mtDNA copies per cell. mtDNA copies per cell decreased with an increasing number of superovulation cycles (Fig. 1C). The results showed that the mean mtDNA copies per cell in R1 (2813.9 ± 648.7) was slightly higher than that in R3 (2034.2 ± 318.0, P = 0.58) and R5 (1458.4 ± 106.9, P = 0.42, Fig. 1C). The average mtDNA copies per cell in R3 was also slightly higher than that in R5 (P = 0.75, Fig. 1C).

### Alterations in DNA methylation level and mRNA expression of *PolgA* in cumulus cells

**Figure 2:**
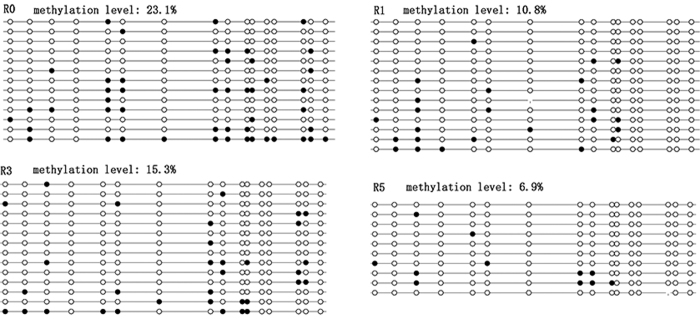
Methylation levels of PolgA in cumulus cells. The methylation level on exon 2 of *PolgA* in cumulus cells was evaluated by bisulfite sequencing. Black circle, methylated sites; white circle, unmethylated sites; no circle, lost sites; R0 (n = 8), natural estrus cycle; R1 (n = 12), one superovulation; R3 (n = 12), three superovulations; R5 (n = 12), five superovulations. The number represents the methylation level.

We further investigated the DNA methylation level and mRNA expression of *PolgA*. The methylation level of *PolgA* in exon 2 in the natural estrus cycle (R0) was similar to that in R3 (P = 0.07). However, the methylation level of *PolgA* in R0 was significantly higher than those in R1 (P = 0.001) and R5 (P < 0.001, Fig. 2). The DNA methylation level of *PolgA* in exon 2 in R1 was slightly higher than that in R5 (P = 0.13), and the methylation level of *PolgA* in R1 was slightly lower than that in R3 (P = 0.202). However, compared to R1, there was no significant difference in R3 and R5. In R5, the methylation level of *PolgA* was significantly lower than that in R3 (Fig. 2, P = 0.011).

**Figure 3:**
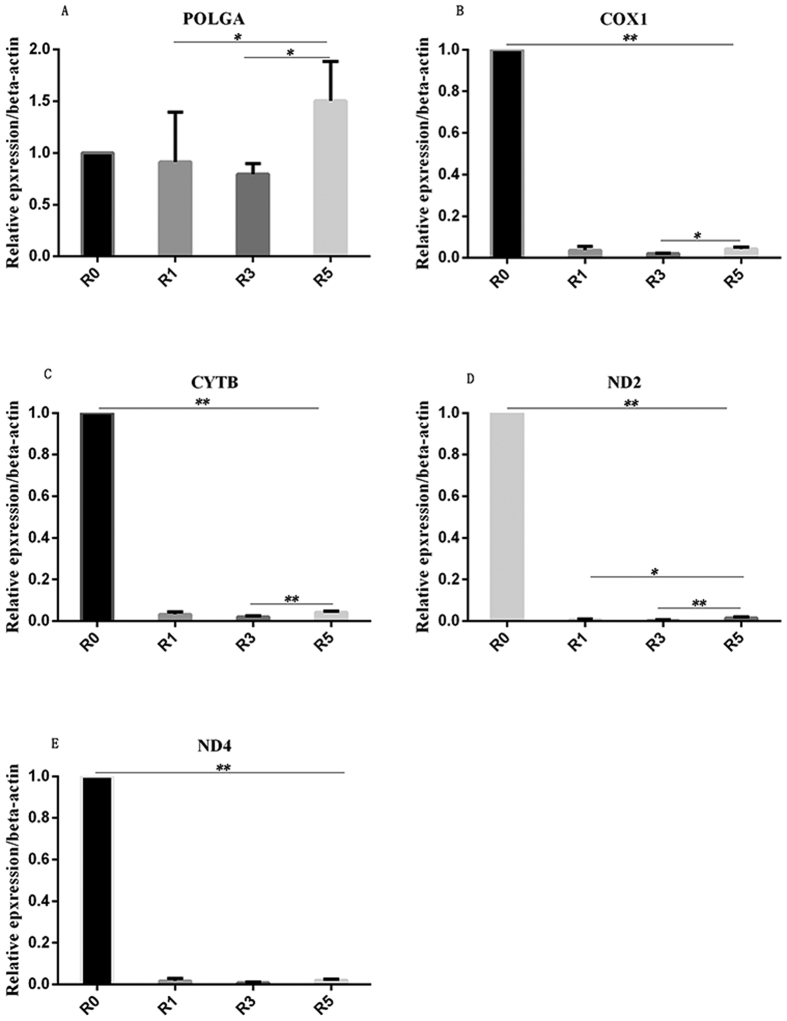
Relative expression of genes in cumulus cells. Gene expression was evaluated by qRT-PCR with β-actin used as a house-keeping gene. The relative expression of POLGA is shown in (**A**) COX1, CYTB, ND2, and ND4 are shown in (**B**–**E**), respectively. For each gene, 12 mice were used. *Presents P < 0.05; **presents P < 0.01; R0: natural estrus cycle; R1 (n = 12), one superovulation; R3 (n = 12), three superovulations; R5 (n = 12), five superovulations.

We also compared the expression in R0 with that in R1, R3 and R5. The expression in R1, R3, and R5 was similar to that in R0 cumulus cells, showing no significant differences. The results revealed decreased expression of POLGA in R3 compared to that in R1, but the difference was not significant (P = 0.655, Fig. 3A). However, we found that the expression of POLGA in R5 was higher than that in R1 (P = 0.048) and R3 (P = 0.024, Fig. 3A). The trend for the changes in *PolgA* expression between R1, R3, and R5 coincided with the methylation levels.

### Effects of repeated superovulation on relative expression of mitochondrial-encoded genes in cumulus cells

Further evaluation in cumulus cells showed that COX1 expression (Fig. 3B) in R1, R3, and R5 was significantly lower than that in R0 (P < 0.001, Fig. 3). COX1 expression in R3 was significantly lower than that in R5 (P = 0.032), but there was no significant difference between R1 and R5 (P = 0.487). For CYTB, expression in R3 was significantly lower than that in R5 (P = 0.006). In R5, CYTB expression was slightly higher than that in R1 (P = 0.135, Fig. 3C). ND2 expression in R5 was higher than that in R1 (P = 0.02) and R3 (P = 0.005, Fig. 3D). However, the expression of ND4 in R1, R3, and R5 was similar (P > 0.05, Fig. 3E).

## Discussion

A previous study showed that repeated superovulation resulted in altered mitochondrial distribution, aggregation of the Golgi apparatus, and expression of octamer-binding transcription factor (Oct4) in oocytes^14^. Another study demonstrated that repeated superovulation altered the expression of many proteins in rhesus monkeys^13^. In humans, repeated superovulation increases mitochondrial mutation in the ovaries^12^. However, few studies have focused on the effects of repeated superovulation on the mitochondria of cumulus cells. In the present study, we found that repeated superovulation altered the number of mtDNA copies per cumulus cell in mice. This indicates that the mtDNA copy number in cumulus cells is decreased by repeated superovulation.

mtDNA replication relies on nuclear-encoded factors that are translocated to mitochondria^21^. The two key factors for mtDNA replication are mitochondrial transcription factor A and the mitochondrial-specific DNA polymerase γ^21^,^22^, which is the only DNA polymerase localized within the mitochondria^23^,^24^. Mammalian polymerase γ is composed of one catalytic subunit (POLGA) and two accessary subunits (POLGB)^25^. The expression of POLGA is associated with mtDNA copy number^18^,^26^. In the present study, we evaluated the expression of POLGA in cumulus cells. The mRNA expression of *PolgA* in R1, R3, and R5 was similar to that in R0, but the expression of *PolgA* in R5 was significantly higher than that in R1. Another study found that POLGA expression was tissue-specific and is regulated by the DNA methylation level in exon 2 of *PolgA*^20^. Thus, we investigated the methylation level of *PolgA* in cumulus cells. We found that the methylation level in R3 was slightly higher than that in R1 and R5. The methylation level of *PolgA* in R1 was higher than that in R5, but the difference was not statistically significant. Although our results indicate that the altered trend in the expression of *PolgA* coincided with that of the methylation level, the change in the expression level of *PolgA* is contradicted by the mtDNA copy number. Kelly *et al*. observed low methylation (<10%) of *PolgA* in oocytes, blastocysts, and embryonic stem cells^20^. They also found that the mtDNA copy number is not associated with methylation level and *PolgA* expression in embryonic stem cells and other tissue-specific cells^20^. Our results also indicate that *PolgA* (<20%) showed a low methylation level in cumulus cells, and the mtDNA number may not be associated with the expression of *PolgA*.

Cumulus cells play a key role in oocyte maturation and the quality of oocyte and embryonic development because most metabolites and energy are supplied by cumulus cells during oogenesis^27^,^28^. ATP can be directly transmitted to the oocyte from cumulus cells via the gap junction^29^,^30^. ATP is mainly produced by the mitochondria in cumulus cells. Mammalian mtDNA encodes 13 polypeptides of the electron transfer chain, 22 tRNA and 2 rRNA^31^, which directly or indirectly contribute to ATP production through the oxidative phosphorylation pathway. Thus, the expression of genes encoded by mtDNA may be affected by repeated superovulation in cumulus cells. In this study, we investigated the relative expression of COX1, CYTB, ND2, and ND4 in cumulus cells. Our results showed that the relative expression of these genes in R3 was lower than that in R1 and R5, but was higher in R5 than in R1 and R3. The altered trend corresponds to the changes in *PolgA*. A previous study demonstrated that the competence of ovulation and fertilization of the oocyte was not compromised when the mtDNA copy number was reduced to 11% of that in the controls^32^. When embryos begin with fewer than 50,000 mtDNA copies, post-implantation development is impeded^32^. This indicates that there is a threshold level of functional mitochondria required for normal development. Thus, a similar threshold of functional mitochondria also exists in cumulus cells. When the mtDNA copies are close to the threshold in cumulus cells, the expression of genes involved in ATP synthesis may increase to support oocyte maturation. This may explain the increased expression of COX1, CYTB, ND2, and ND4 in cumulus cells of R5. A study showed that the expression of COX1 is higher in oocytes with compacted cumulus than in oocytes with expanded cumulus^33^. When cell lines containing cumulus cells are cultured in serum-starvation medium, mitochondrial viability is dramatically higher than that in cells cultured under control conditions^34^. This indirectly confirms that the increased expression of genes can be induced by decreasing mtDNA copies in cumulus cells. The altered methylation level may also explain the alteration of these genes. A previous study suggested that the decrease in expression of mtDNA-encoding genes was associated with increased methylation levels of these genes^35^. In this study, we found that the change in *PolgA* expression was correlated to the change in the methylation level of exon 2. The expression of other genes was similar to that of *PolgA*. Thus, the altered methylation level may contribute to changing gene expression.

There were some limitations to the present study. We did not completely explain how repeated superovulation affects mitochondrial function in cumulus cells. Kalthur *et al*. found that multiple superovulation altered the distribution of cytoplasmic organelles, destroyed the integrity of the spindle, altered the expression of *Oct4*, and increased reactive oxygen species accumulation in oocytes^14^. Therefore, we did not determine whether oocyte quality was affected by repeated superovulation.

In summary, we found that repeated superovulation altered mtDNA copies in mouse cumulus cells. The alteration in mtDNA copies per cell may be not associated with decreased DNA methylation levels on exon 2 of *PolgA*. The expression of genes encoded by mtDNA was also changed by repeated superovulation in cumulus cells. Therefore, repeated superovulation adversely affected mitochondrial function in cumulus cells, which may reduce oocyte quality and delay embryonic development.

## Materials and Methods

Animal care and use were conducted in accordance with the guideline of Qingdao Agricultural University, China. Mice were housed in a temperature-controlled room with proper darkness-light cycles and fed a regular diet. All experiments and the study protocol were approved by the Ethics Committee of Qingdao Agricultural University, China.

### Superovulation

Female ICR mice (5 weeks of age) were purchased from the Center of Experimental Animals of Qingdao and fed in a temperature- and humidity-controlled room at a light cycle of 12 h light and 12 h dark. Diet and water were supplied *ad libitum*.

Female CD1 mice were divided into four groups: (a) natural estrus cycle (R0); (a) intraperitoneal injection with 8 IU PMSG and 8 IU hCG 48 h later (R1); (b) repeated intraperitoneal injection with 8 IU PMSG and 8 IU hCG 48 h later three times at 7-day intervals (R3); (c) repeated intraperitoneal injection with 8 IU PMSG and 8 IU hCG 48 h later five times at 7-day intervals (R5).

### Cumulus cell collection

After intraperitoneal injection of human chorionic gonadotropin (hCG) 13–14 h later, female mice were sacrificed by cervical dislocation, and the cumulus-oocyte complex was collected from the oviduct ampulla. Cumulus cells and oocyte were separated using 1 mg/mL hyaluronidase. Cumulus cells were collected in 1.5-mL Eppendorf tubes and centrifuged at 10,000 rpm for 10 min. The supernatant was discarded, and the sediment was washed three times with PBS. The samples were stored at −20 °C until use. To collect cumulus cells of R0, proestrus mice were selected. Approximately 15 h later, the cumulus-oocyte complexes were collected from the oviduct ampulla, and cumulus cells were retrieved as previously described.

### Total DNA and RNA purification from cumulus cells

Total DNA and RNA of cumulus cells were extracted by using DNA extracted kit (Sangon Biotech, Beijing) and EZ-10 Spin Column Total RNA Isolation Kit (Sangon Biotech, Beijing) according to the manufacturer’s instruction. Total DNA and RNA were stored at −20 °C and −80 °C, respectively.

### Quantitative real-time PCR (qRT-PCR)

First-strand of cDNA was synthesized using the FastQuantRT Kit (TianGen, Beijing, China) according to the manufacturer’s instructions. Briefly, genomic DNA was removed using DNase I at 42 °C. Next, 5 μL total RNA was added to reverse transcription mix and incubated at 42 °C for 15 min. The enzyme was inactivated at 95 °C for 3 min and then incubated on ice. The synthesized cDNA was used as a template for qRT-PCR. Primers are shown in Table 1. qRT-PCR was carried out using the Roche LightCycler 480II (Roche Diagnostics, Basel, Switzerland). Βeta-actin was used as a control gene, and relative expression was calculated using the 2^−∆∆Ct^ method.

mtDNA copies per cell was evaluated by real-time PCR as previously described^36^. Briefly, the mtDNA fragment and β-actin were amplified by PCR, and the products of PCR were ligated into the T-vector. To generate the standard curve, 10-fold serial dilutions of purified plasmid standard DNA were used. All measurements were performed in triplicate. Total DNA extracted from cumulus cells was used to determine the mtDNA copy number per cell, which was calculated using the follow formula:


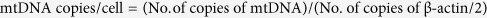


### DNA bisulfite and sequencing

Total DNA was treated using the EZ DNA Methylation-Direct kit (Zymo Research, Irvine, CA, USA) according to the manufacturer’s instructions. Modified DNA was used as template for PCR. The CpG island in exon 2 of *PolgA* was amplified using nested PCR, and the primers are shown in Table 1. The amplified PCR products were ligated into the T-vector (Takara, Shiga, Japan) and sequenced (GENEWIZ, Shanghai, China).

### Statistical analysis

The expression of genes and mtDNA copies per cell were represented as the mean ± SD. Differences were evaluated by one-way analysis of variance. The methylation level was tested by Chi-square test. If the P value was <0.05, the difference between groups was considered significant.

## Additional Information

**How to cite this article**: Xie, J.-K. *et al*. Repeated superovulation may affect mitochondrial functions of cumulus cells in mice. *Sci. Rep*. **6**, 31368; doi: 10.1038/srep31368 (2016).

## Figures and Tables

**Table 1:** Oligo sequences used in real-time PCR and bisulfite sequencing (BS).

	Genes	Forward	Reverse
Real-time PCR	mtDNA fragment	CGACCTCGATGTTGGATCA	AGAGGATTTGAACCTCTGG
	PolgA	GAGCCTGCCTTACTTGGAGG	GGCTGCACCAGGAATACCA
	ND2	CCATTCCACTTCTGATTACC	GTCATGTAAGAAGAATAAGTCC
	CytB	ATTCCTTCATGTCGGACGAG	ACTGAGAAGCCCCCTCAAAT
	Cox1	TTTTCAGGCTTCACCCTAGATGA	CCTACGAATATGATGGCGAAGTG
	ND4	CCAGCCTAACACTTCTATG	GGCTAGCTATTAATATTAGTGGC
	β-actin	TATTGGCAACGAGCGGTTCC	GGCATAGAGGTCTTTACGGATGTC
BS	Out-PolgA	TAAGTAAGGTAGGTTTTGTTTTTGG	AAAACAAACATAATAAAACCTAT
	Inner-PolgA	GGAAGTGTTGGTTTAGGTTGTTTT	AACATAATAAAACCTATTTCAC
